# Association between Physical Fitness Index and Psychological Symptoms in Chinese Children and Adolescents

**DOI:** 10.3390/children9091286

**Published:** 2022-08-26

**Authors:** Jinkui Lu, Hao Sun, Jianfeng Zhou, Jianping Xiong

**Affiliations:** 1 School of Physical Education, Shangrao Normal University, Shangrao 334000, China; 2 Physical Education College, Jiangxi Normal University, Nanchang 330022, China; 3 School of Sports, Jiangxi University of Finance and Economics, Nanchang 330013, China

**Keywords:** mental health, PFI, children and adolescents, association analysis, cross-sectional survey

## Abstract

The aim of this study was to determine the relationship between different physical fitness indices (PFIs) and psychological symptoms and each dimension (emotional symptoms, behavioral symptoms, social adaptation difficulties) of Chinese children and adolescents. Methods: A total of 7199 children and adolescents aged 13–18 in Jiangxi Province, China, were tested for grip strength, standing long jump, sit-ups, sit and reach, repeated straddling, 50 m run, 20 m shuttle run test (20 m SRT) items. The physical fitness indicators were standardized, converted to Z score and added up to obtain the PFI, and the self-assessment of the psychological section of the multidimensional sub-health questionnaire of adolescents (MSQA) to test the psychological symptoms, using the chi-square test to determine the psychological symptoms of different types of children and adolescents and binary logistic regression analysis to determine the association between psychological symptoms and different PFI grades. Results: The higher the PFI of Chinese children and adolescents, the lower the detection rate of psychological symptoms, emotional symptoms and social adaptation difficulties, from 25.0% to 18.4%, 31.3% to 25.7% and 20.1% to 14.4%, respectively. These results were statistically significant (χ^2^ = 14.073, 9.332, 12.183, *p* < 0.05). Taking the high-grade PFI as a reference, binary logistic regression analysis was performed. Generally, compared with the high-grade PFI, children and adolescents with a low-grade PFI (*OR* = 1.476, 95% CI: 1.200–1.814) or medium-grade PFI (*OR* = 1.195, 95% CI: 1.010–1.413) had a higher risk of psychological symptoms (*p* < 0.05). Conclusions: The lower the PFI of Chinese children and adolescents, the higher the detection rate of psychological symptoms, showing a negative correlation. In the future, measures should be taken to improve the physical fitness level of children and adolescents in order to reduce the incidence of psychological symptoms and promote the healthy development of children and adolescents.

## 1. Introduction

As the economy continues to grow, and the level of medical care and technology are improved, people’s quality of life is greatly improved. However, this also leads to many adverse effects on human health, such as the increase in obesity [1], screen time [2] and the detection rate of psychological symptoms as well as decreased physical activity [3], resulting in a continuous increase in the proportion of people with psychological symptoms. Adolescence is a transitional stage from childhood to adulthood in which physiological development gradually matures, but psychological development relatively lags behind, causing a serious imbalance [4,5]. In addition, this stage is affected by complex changes such as interpersonal relationships and academic pressure, which can easily lead to various psychological symptoms [6,7,8]. Research shows that the proportion of psychological symptoms in children and adolescents is increasing year by year and is much higher than that of other age groups [9]. Epidemiological survey data show that 10%~20% of adolescents worldwide are affected by different psychological symptoms, accounting for a large part of the global disease burden [10], and 50% of psychological symptoms in adulthood are caused by childhood, indicating a trajectory effect [11]. Studies have confirmed [12] that the appearance of psychological symptoms in children and adolescents leads to the decline of learning and self-care ability and has a serious negative impact on the healthy growth of children, adolescents and their families. Other studies have shown that [13] psychological symptoms are the initial stage of mental disorders, which will develop without timely intervention.

Past research has shown that the world is facing a serious issue in the prevention and control of psychological symptoms. About 6 million people in the United States suffer from psychological symptoms every year, along with 35% of the population in Japan and 37% of the population in Australia [14]. Seventy percent of the population in China suffers from various psychological symptoms, with children and adolescents making up the largest proportion [15]. There are also studies showing that the proportion of Chinese children and adolescents who suffer from various psychological symptoms affecting their daily life and study is increasing [16,17]. In addition, the self-injury and suicide behaviors of children and adolescents due to various psychological symptoms are also increasing, which poses a serious threat to the healthy development of Chinese children and adolescents [18,19]. Another survey shows that about 30 million children and adolescents in China are experiencing psychological symptoms, and the detection rate is increasing year by year, with a higher incidence among younger children [19,20]. It can be seen from these findings that children and adolescents in China are facing a severe situation of continuous increase in psychological symptoms.

It is an indisputable fact that modern lifestyle changes have led to decreased physical fitness and physical activity and increased screen time among children and adolescents [21]. Studies have shown that among various factors leading to death, death due to reduced physical fitness and reduced exercise time has become the fourth most prevalent [22]. Research also shows that people with higher muscle strength have improved psychological symptoms, and the two are positively correlated [23,24]. A survey conducted by Tao et al. [25] of 5453 middle school students in China showed that low- to moderate-intensity physical activity was a protective factor for depression in children and adolescents. Research showed that there was a negative correlation between physical fitness levels and psychological symptom indicators of boys and girls [25,26]. An increase in the weekly physical exercise time of 1 h would reduce the proportion of the number of people suffering from depression by 8% [27]. A 4-year follow-up study on children and adolescents aged 14~24 showed that adequately ensuring physical exercise time and promoting physical fitness can reduce the occurrence of depression, anxiety and other adverse psychological problems [27]. There are also studies showing that the improvement of cardiorespiratory fitness level plays a positive role in reducing the occurrence of anxiety and depression, as active physical exercise can improve body shape, enhance self-confidence and thus reduce the occurrence of psychological symptoms [28]. Active physical exercise can also increase the secretion of endorphins and dopamine in the brain, which can promote physical and mental pleasure and improve psychological symptoms [27,29]. Regular physical exercise can effectively reduce the risk of various diseases, such as hypertension, type 2 diabetes, depression, anxiety and various types of cancer and coronary heart disease. The reduction in physical fitness is an important reason for the continued decline in psychological symptoms [29].

Combining the above studies on physical fitness, physical exercise and psychological symptoms by scholars from various countries, it can be seen that there is an association between them [30,31,32]. However, most of the previous studies on the psychological symptoms of children and adolescents have used the clinical diagnostic scale of “mental disease” to conduct research and evaluation, mainly with the dimensions of anxiety, emotional disorders, depression and hostility [33]. These scales are suitable for use in the clinical, psychiatric and psychological counseling fields but cannot effectively screen children and adolescents with psychological symptoms, so early intervention and prevention of psychological symptoms cannot be carried out. Furthermore, previous studies have mainly focused on the relationship between physical fitness programs and psychological symptoms, while the relationship between PFI and psychological symptoms of children and adolescents has been less studied [33,34]. In view of this, this study investigated the psychological symptoms of 7199 Chinese children and adolescents aged 13–18 using the MSQA scale and tested seven physical fitness indicators to determine the relationship between different PFIs and psychological symptoms.

## 2. Methods

### 2.1. Data Source and Participants

The data collection for this study was carried out with the help of the National Student Physical Health Standard (NSPHS) test. The NSPHS test is organized and implemented by the Chinese Ministry of Education to investigate the physical fitness of school students across the country [35].

**Figure 1:**
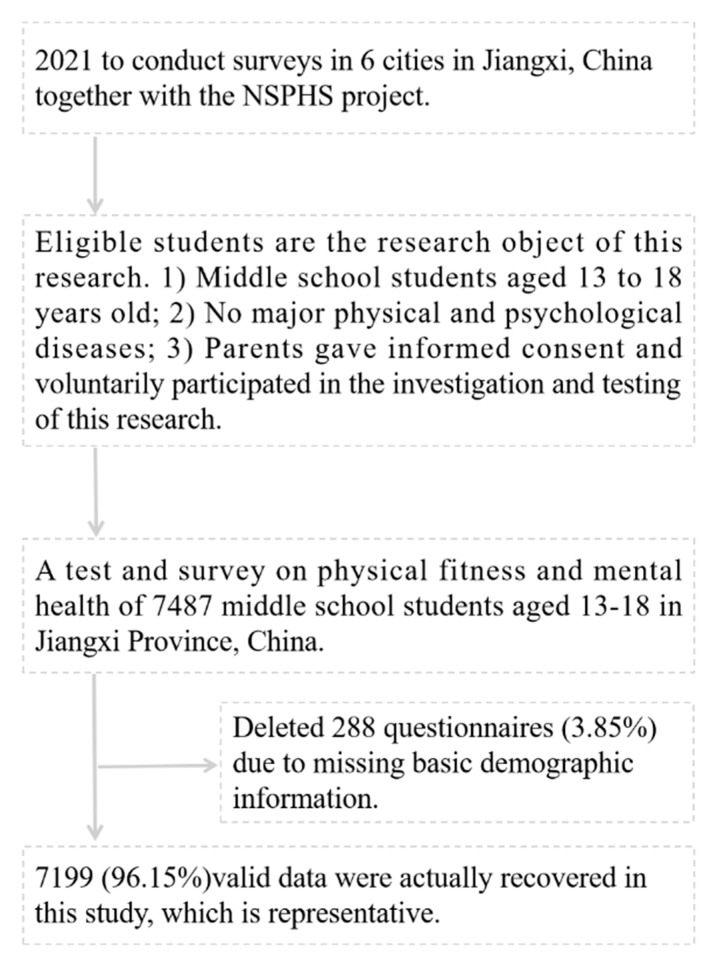
The specific sampling process of the participants.

This study investigated 6 age groups from 13 to 18 years old. Surveys were conducted in 6 cities in Jiangxi, and it was concluded that the sample size should be 6660 people. After the investigation, after removing 288 (3.85%) responses with missing basic demographic information, 7199 (96.15%) valid surveys were retained in this study. The specific sampling process of the participants is shown in Figure 1.

This study was approved by the Ethics Committee of Shangrao Normal University (2020R-0125). Written informed consent was obtained from the school, the students and their parents before the investigation. The questionnaire was coded to strictly protect the privacy of students.

### 2.2. Psychological Symptoms

Psychological symptoms were evaluated using the multidimensional sub-health questionnaire of adolescents (MSQA) [36,37,38,39,40]. The MSQA consists of 39 items, which are divided into 3 dimensions: emotional symptoms, behavioral symptoms and social adaptation difficulties. Emotional symptoms were tested with 17 items, behavioral symptoms were tested with 9 items and social adaptation difficulties were tested with 13 items. Each item is scored on a 6-level scale, and the subjects choose the corresponding duration according to their own conditions: none or less than 1 week = 1, more than 1 week = 2, more than 2 weeks = 3, more than 1 month = 4, more than 2 months = 5, more than 3 months = 6. The results were reclassified during the statistical analysis of the questionnaire, as the responses of more than 1 month, more than 2 months and more than 3 months were recorded as 1, and the others were recorded as 0, and psychological symptoms were calculated as the sum of the scores of all 39 items. The three dimension scores, namely emotional symptoms, behavioral symptoms and social adaptation difficulties, were calculated separately according to the items.

According to the provisions of the “National Norm Development of the Multidimensional Assessment Questionnaire for Adolescents’ Sub-health” [37,38,39,40], the 90th percentile was used as the demarcation value of the psychological symptoms of adolescent students of all ages and as the criterion for judging the psychological symptoms of children and adolescents. That is, the values for emotional symptoms, behavioral symptoms, social adaptation difficulties and psychological symptoms were ≥3, ≥1, ≥4 and ≥8, respectively.

The test–retest correlation coefficient of the scale was 0.868, the Cronbach α coefficient was 0.957 and the split-half reliability was 0.942. The self-rating symptom scale (SCL-90) and the Cornell Medical Index (CMI) questionnaire were used as the criteria, and the criterion-related validity was 0.636 and 0.649, respectively [39].

### 2.3. Physical Fitness Index

The physical fitness index (PFI) can comprehensively reflect the development level of physical fitness of children and adolescents to a certain extent and has been applied and recognized in many studies [41,42]. The scores of the 7 physical fitness test indicators were standardized into groups according to gender and age, and the Z score was calculated: Z score = (measured value of each physical fitness indicator − average value of each physical fitness indicator in each group)/standard deviation of each physical fitness index in each group. The PFI is obtained by adding up the Z scores of each physical fitness index and taking the opposite number for the Z score of the 50 m run because the higher the Z score, the lower the subject’s performance.

PFI = Z _grip strength_ + Z _standing long jump_ + Z _sit-ups_ + Z_sit and reach_ + Z _repeated straddling_ + Z _20 m SRT_ − Z _50 m run_.

According to the PFI percentiles of different gender and age groups, PFI was stratified [43] and divided into three grades: low, medium and high: low PFI < P15, medium P15 ≤ PFI < P85 and high PFI ≤ P85.

The physical fitness test includes 7 items, namely grip strength, standing long jump, sit-ups, sit and reach, repeated straddling, 50 m run and 20 m SRT.

Grip strength: The subjects were required to use their left or right hand to perform the electronic grip dynamometer test. During the test, the subjects were required to relax their hands naturally and perform two grip strength tests with maximum effort. The highest result, accurate to 0.1 kg, was recorded.

Standing long jump: The subjects stood naturally with their feet apart behind the jumping line. Participants jumped with both feet, with no step-by-step jumping. The distance from the subject to the closest touchdown point was measured. Each subject jumped twice, and the best score was recorded. The test results are accurate to 0.1 cm.

Sit-ups: Before the test, the staff prepared the mat and stopwatch. The subjects lay in the supine position on the mat, with relaxed bodies, hands are crossed on chest, knees bent at 60–90 degrees, and feet flat on the ground. The assistant pressed the subjects’ ankles with both hands to immobilize their lower limbs. When the subjects heard the “start” command, they began to do sit-ups, with their elbows touching or exceeding their knees, and then quickly returned to the initial lying supine position with their back touching the mat. The above actions were repeated as much as possible. The number of sit-ups (where the elbows touched the knees or the thighs) was recorded.

Sit and reach: Before the test, the inspector prepared the instrument and cushion, selected a flat ground and checked the working state of the sit and reach instrument. The subject sat upright on the mat, with the head, back and buttocks close to the wall, and the feet were placed under the instrument, but the angle of the feet was not fixed. The subject extended their arms shoulder-width apart, placed their palms on the test instrument board, expanded their chest and kept their hands close to the instrument board with their elbows stretched forward and back straight. Initially in a sitting position, the subject flexed and stretched forward and slowly pushed the instrument in the forward direction. When the body flexed and stretched forward to the maximum, the data were recorded, and the subject left the test board using both hands to end the test. The movement distance of the instrument from the initial position to the maximum flexion and extension was recorded. Measurements were in centimeters (cm) with one decimal place. Each subject was tested twice, with the best result recorded.

Repeated straddling: Two parallel lines were drawn at a distance of 100 cm from the central line, for a total of 3 lines. The subjects stood with their feet open on the central line. When they heard the “start” command, they crossed the horizontal line in the order of right → middle → left middle. Participants were instructed not to jump with both feet. The number of completed actions within 20s was recorded [44].

50 m run: Before the test, several straight runways were drawn with a length of 50 m and a width of 1.22 m on the flat ground with clear runway lines. The timekeeper stood on the side of the finish line, started timing when the starting flag was waving and stopped when the subject’s chest reached the vertical plane of the finish line. The time score was accurate to 0.1s.

20 m SRT: This test involves the subject running back and forth between 2 lines at a distance of 20 m. Each 20 m completed was recorded as 1 lap (time). The running speed was indicated by music, and the initial speed was 8.0 km/h, the speed in the second minute was 9.0 km/h and the speed was increased by one speed level every minute, that is, 0.5 km/h each lap. The subject endeavored to complete the running speed level. The test ended when they could not follow the rhythm to reach the 20 m end line for two consecutive times, and the number of laps completed was recorded [45,46].

### 2.4. Statistical Analysis

In the basic condition part, each physical fitness index of 7199 children and adolescents was expressed as mean ± standard deviation (M ± SD), and the detection rate of psychological symptoms and each dimension was expressed as a percentage of classification.

The chi-square test was used to compare the detection rates of different categories, namely psychological symptoms and dimensions (emotional symptoms, behavioral symptoms, and social adaptation difficulties) of children and adolescents with different PFIs. The relationship between different levels of PFI and various dimensions of psychological symptoms in children and adolescents was analyzed with binary logistic regression analysis and interaction effect analysis, and the OR value, 95% confidence interval and *p* value were obtained; *p* < 0.05 was regarded as a statistically significant difference. Our research hypothesis is that children and adolescents with higher PFI have a lower detection rate of psychological symptoms.

Data analysis was performed with SPSS 25.0 software, graphs were produced with GraphPad Prism and the statistical significance level was set at 0.05.

## 3. Results

The results showed that the average age of the 7199 participating Chinese children and adolescents was (15.50 ± 1.71) years old. All physical fitness indicators for boys were higher than those of girls except for the sit and reach test. Low, middle and high PFIs were segmented by percentiles, so the proportion of males and females was the same at each level. The detection rate of psychological symptoms and each dimension (emotional symptoms, behavioral symptoms, social adaptation difficulties) in males was higher than that in females (Table 1).

The results of our study showed that the higher the PFI of Chinese children and adolescent boys, the lower the detection rate of psychological symptoms and social adaptation difficulties, and there was statistical significance (χ2 = 8.106, 7.065, *p* < 0.05). The higher the PFI of girls, the lower the detection rate of psychological symptoms and social adaptation difficulties, and there was statistical significance (χ2 = 10.922, 6.172, *p* < 0.05). In general, the higher the PFI of Chinese children and adolescents, the lower the detection rate of psychological symptoms, emotional symptoms and social adaptation difficulties, from 25.0% to 18.4%, 31.3% to 25.7% and 20.1% to 14.4%, respectively, and there was statistical significance (χ2 = 14.073, 9.332, 12.183, *p* < 0.05) (Table 2).

Using the psychological symptoms of children and adolescents in the high-grade PFI group as reference, binary logistic regression analysis was performed. Boys with a low-grade (OR = 1.485, 95% CI: 1.104–1.998) or middle-grade (OR = 1.375, 95% CI: 1.082–1.749) PFI had a higher risk of psychological symptoms, with statistical significance (*p* < 0.05). Psychological symptoms were risk factors for low grades of PFI in girls (OR = 1.467, 95% CI: 1.100–1.957) compared with high grades of PFI (*p* < 0.05). Overall, compared with the high-grade PFI, children and adolescents with a low-grade (OR = 1.476, 95% CI: 1.200–1.814) or medium-grade PFI (OR = 1.195, 95% CI: 1.010–1.413) were more likely to develop psychological symptoms (*p* < 0.05) (Table 3).

**Figure 2:**
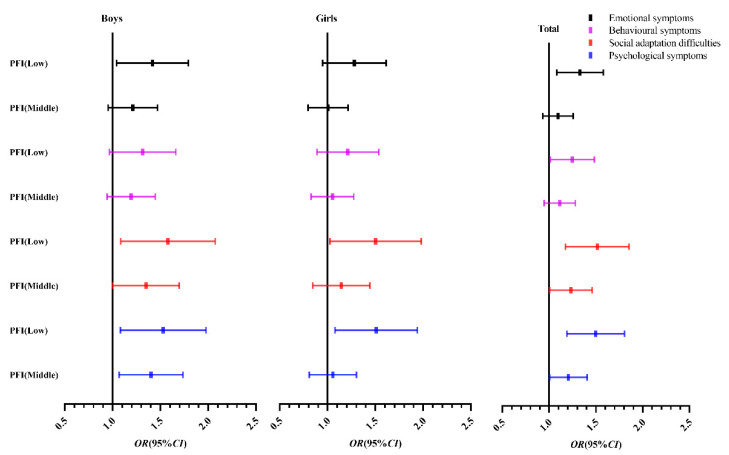
Binary logistic regression analysis *OR* value (95% CI) of psychological symptoms of Chinese children and adolescents. Note: The high grade of PFI is 1.0; OR (95% CI), odds ratio (95% confidence interval).

Figure 2 shows that compared with the high-grade PFI group, the middle-grade PFI and especially the low-grade PFI group are more inclined to the right; that is, the risk of psychological symptoms is higher.

## 4. Discussion

The results of our study showed that the detection rate of psychological symptoms in Chinese children and adolescents was 21.4%, which was lower than the results of the psychological symptoms survey involving middle school students in nine cities in China (36.73%) [47]. A survey of middle school students showed that the detection rate of psychological symptoms was 64.1% for middle school students and 68.9% for high school students [48], lower than the results of this study. The reason for this difference is related to the differences in the selected regions, age groups and ethnic groups of the surveyed subjects and the differing research length. For example, in recent years, China has increased investment in the education of children and adolescents’ mental health and strengthened teachers, which is also an important reason for the low proportion of psychological symptoms [49]. In addition, there are differences in the evaluation criteria or questionnaires of psychological symptoms used in different studies, which is also an important reason for the large differences in the results of different studies [47,48]. Although the detection rate of psychological symptoms in children and adolescents continues to decrease, it is still at a high level. Therefore, social attention is needed to continuously reduce the detection rate of psychological symptoms in children and adolescents. Although the results are different, it is still vital to conduct research on the influencing factors and interventions of psychological symptoms in children and adolescents, and the results also provide theoretical support for the intervention aimed at children and adolescents with psychological symptoms. Our research also shows that the detection rate of different dimensions of psychological symptoms also differs between genders. The detection rate of boys is higher than that of girls in emotional symptoms, behavioral symptoms, social adaptation difficulties and psychological symptoms. The reason may be related to the congenital personality differences between boys and girls. Society and the family endow boys with greater responsibilities and expectations, resulting in a higher detection rate of psychological symptoms.

In this study, the PFI was obtained by standardizing the Z score of nine physical fitness items and divided into high-, medium- and low-level groups according to the percentile. The higher the PFI, the lower the detection rate of each dimension of psychological symptoms in children and adolescents, and these findings were statistically significant (*p* < 0.01). Relevant studies have shown that physical activity plays a positive role in improving psychological symptoms [50,51,52]. Other studies have shown that active participation in physical exercise and a good level of physical fitness play a positive role in promoting the mental health of children and adolescents, and mentally healthy children and adolescents are also more willing to improve their athletic ability through physical exercise [53,54]. The Matute-Llorente [55] study showed that the longer the daily exercise time in children and adolescents, the higher the level of cardiorespiratory fitness. The Aires [56] study also confirmed that the longer children and adolescents participate in exercise every day, the higher their cardiorespiratory fitness level. Similarly, studies have confirmed that the higher the level of cardiorespiratory fitness, the lower the detection rate of psychological symptoms in children and adolescents, which further confirms the close relationship between physical fitness level and psychological symptoms [57,58,59]. They also confirmed the conclusion that children and adolescents with higher PFI had lower detection rate of psychological symptoms in this study. Our study also showed that the influence of PFI on psychological symptoms in boys was significantly higher than that in girls, especially when the PFI was at a middle level. The reason may be that boys prefer physical exercise, their physical fitness is better and the impact on psychological symptoms is more obvious. Conversely, girls’ participation in exercise is lower, so the effect on psychological symptoms is not significant.

Our research also has certain advantages. The sample size of the study is large, leading to representative results. The use of PFI, which comprehensively reflects physical fitness, can also more accurately analyze the relationship between physical fitness and psychological symptoms. Our research also has some shortcomings. First, this study is a cross-sectional study, which can determine the association between physical fitness and psychological symptoms but not the causal relationship between the two. Prospective cohort studies should be conducted in the future on this topic. Second, the sample population of this study was located in Jiangxi, China, and the survey area was thus limited. The scope of the survey should be expanded in the future to support these findings. Third, the covariates in the investigation and analysis of this study were limited. The investigation of covariates, such as sleep quality, physical activity time and screen time, should be added in future studies to verify our results.

Based on the above studies, it can be seen that PFI is closely related to the detection rate of psychological symptoms in Chinese children and adolescents. Reasonable arrangements for active participation in physical exercise and improving physical fitness play a positive role in preventing the occurrence of psychological symptoms in children and adolescents. Of course, the reduction in psychological symptoms in children and adolescents cannot be achieved solely by the improvement of physical fitness level. Although a large number of theories and practices have confirmed that physical fitness improvement can cultivate good mentality and values in children and adolescents, thereby promoting a good life and habits and the healthy development of the body and mind, the development of children and adolescents’ mental health is affected by multiple factors. We should also start from the aspects of children and adolescents’ health education, family guidance and social environment and jointly promote the physical and mental health of children and adolescents.

## 5. Conclusions

By testing seven physical fitness items of 7199 children and adolescents aged 13–18 in China, the physical fitness indicators were standardized and converted into Z scores, and the PFI was obtained after adding them up. The higher the PFI, the lower the detection rate of psychological symptoms, emotional symptoms, behavioral symptoms and social adaptation difficulties, showing a negative correlation between PFI and psychological symptoms. In addition, compared with girls, boys’ PFI had a more significant effect on psychological symptoms. In the future, the physical fitness level of Chinese children and adolescents should be improved, and the detection rate of psychological symptoms in children and adolescents should be reduced so as to jointly promote the physical and mental development of Chinese children and adolescents.

## Figures and Tables

**Table 1:** Physical fitness indicators and psychological symptoms of Chinese children and adolescents.

Classification	Total	Boys	Girls
N	7199	3600	3599
Physical fitness indicators (M ± SD)			
Grip strength (kg)	31.45 ± 9.99	37.05 ± 9.83	25.85 ± 6.36
Standing long jump (cm)	188.24 ± 33.64	209.43 ± 30.15	167.06 ± 21.37
Sit-ups	23.01 ± 6.90	24.80 ± 6.98	21.21 ± 6.33
20 m SRT	36.76 ± 17.17	43.12 ± 18.61	30.40 ± 12.75
Sit and reach (cm)	38.77 ± 11.72	38.12 ± 11.74	39.42 ± 11.66
Repeated straddling	31.54 ± 9.19	33.42 ± 9.72	29.66 ± 8.21
50 m run (s)	8.53 ± 1.26	7.86 ± 1.01	9.21 ± 1.12
PFI, *n* (%)			
Low	1080 (15.0)	540 (15.0)	540 (15.0)
Middle	5040 (70.0)	2520 (70.0)	2520 (70.0)
High	1080 (15.0)	540 (15.0)	539 (15.0)
Psychological symptoms, *n* (%)			
Emotional symptoms	1995 (27.7)	1001 (27.8)	993 (27.6)
Behavioral symptoms	1981 (27.5)	1021 (28.4)	960 (26.7)
Social adaptation difficulties	1236 (17.2)	656 (18.2)	580 (16.1)
Psychological symptoms	1540 (21.4)	797 (22.1)	743 (20.6)

Note: N, sample size; M ± SD, mean ± standard deviation; PFI, physical fitness index; 20 m SRT, 20 m shuttle run test.

**Table 2:** Comparison of detection rates of psychological symptoms in different categories of Chinese children and adolescents (%).

Category		N	Emotional Symptoms	χ2-Value	*p*-Value	Behavioral Symptoms	χ2-Value	*p*-Value	Social Adaptation Difficulties	χ2-Value	*p*-Value	Psychological Symptoms	χ2-Value	*p*-Value
Boys	PFI (Low)	540	167 (30.9)	5.662	0.059	164 (30.4)	3.505	0.173	112 (20.7)	7.065	0.029	130 (24.1)	8.106	0.017
	PFI (Middle)	2520	702 (27.9)			720 (28.6)			465 (18.5)			572 (22.7)		
	PFI (High)	540	132 (24.4)			137 (25.4)			79 (14.6)			95 (17.6)		
Girls	PFI (Low)	540	171 (31.7)	5.234	0.073	156 (28.9)	1.921	0.383	105 (19.4)	6.172	0.046	140 (25.9)	10.922	0.004
	PFI (Middle)	2520	677 (26.9)			661 (26.2)			395 (15.7)			499 (19.8)		
	PFI (High)	539	146 (27.0)			138 (25.6)			77 (14.3)			104 (19.3)		
Total	PFI (Low)	1080	338 (31.3)	9.332	0.009	320 (29.6)	4.724	0.094	217 (20.1)	12.183	0.002	270 (25.0)	14.073	0.001
	PFI (Middle)	5040	1379 (27.4)			1381 (27.4)			860 (17.1)			1071 (21.3)		
	PFI (High)	1079	278 (25.7)			275 (25.5)			156 (14.4)			199 (18.4)		

Note: N, sample size; PFI, physical fitness index.

**Table 3:** Binary logistic regression analysis of psychological symptoms of Chinese children and adolescents in different categories.

Category		Emotional Symptoms			Behavioral Symptoms			Social adaptation Difficulties			Psychological Symptoms		
		*OR* (95% CI)		*p*-Value	*OR* (95% CI)		*p*-Value	*OR* (95% CI)		*p*-Value	*OR* (95% CI)		*p*-Value
Boys	PFI (Low)	1.384	1.059–1.809	0.017	1.283	0.983–1.675	0.067	1.527	1.113–2.096	0.009	1.485	1.104–1.998	0.009
	PFI (Middle)	1.194	0.963–1.479	0.106	1.177	0.952–1.455	0.133	1.320	1.019–1.711	0.035	1.375	1.082–1.749	0.009
	PFI (High)	1.000	1.000		1.000	1.000		1.000	1.000		1.000	1.000	
Girls	PFI (Low)	1.251	0.962–1.626	0.095	1.183	0.905–1.548	0.219	1.451	1.052–2.003	0.023	1.467	1.100–1.957	0.009
	PFI (Middle)	0.991	0.804–1.222	0.935	1.036	0.837–1.281	0.746	1.118	0.858–1.455	0.409	1.035	0.818–1.310	0.774
	PFI (High)	1.000	1.000		1.000	1.000		1.000	1.000		1.000	1.000	
Total	PFI (Low)	1.314	1.089–1.585	0.004	1.233	1.020–1.489	0.030	1.489	1.188–1.866	0.001	1.476	1.200–1.814	0.000
	PFI (Middle)	1.087	0.935–1.262	0.277	1.105	0.951–1.284	0.193	1.219	1.013–1.466	0.036	1.195	1.010–1.413	0.038
	PFI (High)	1.000	1.000		1.000	1.000		1.000	1.000		1.000	1.000	

Note: PFI, physical fitness index; *OR* (95% CI), odds ratio (95% confidence interval).

## Data Availability

To protect the privacy of participants, the questionnaire data will not be disclosed to the public. If necessary, you can contact the corresponding author.
